# Evaluation of a novel real-time PCR assay for the detection, identification and quantification of *Plasmodium* species causing malaria in humans

**DOI:** 10.1186/s12936-021-03842-8

**Published:** 2021-07-12

**Authors:** Kim van Bergen, Toon Stuitje, Robert Akkers, Eric Vermeer, Rob Castel, Theo Mank

**Affiliations:** 1 Result Laboratorium, Albert Schweitzer Hospital, Albert Schweitzerplaats 25, 3300 AK Dordrecht, The Netherlands; 2 MRC Holland, Willem Schoutenstraat 1, 1057 DL Amsterdam, The Netherlands; 3 Streeklaboratorium Voor de Volksgezondheid Kennemerland, Boerhaavelaan 26, 2035 RC Haarlem, The Netherlands

**Keywords:** Malaria, Real-time PCR, Melting curve analysis, Identification and quantification of *Plasmodium* species

## Abstract

**Background:**

The entry of PCR-based techniques into malaria diagnostics has improved the sensitivity and specificity of the detection of *Plasmodium* infections. It has been shown that humans are regularly infected by at least six different *Plasmodium* species. The MC004 real-time PCR assay for malaria diagnosis is a novel single-tube assay that has been developed for the purpose of simultaneously detecting all *Plasmodium* species known to infect humans, and discrimination between *Plasmodium falciparum, Plasmodium vivax, Plasmodium malariae, Plasmodium ovale wallikeri*, *Plasmodium ovale curtisi*, *Plasmodium knowlesi* (including differentiation of three strains) and *Plasmodium cynomolgi* (including differentiation of three strains). Detection and identification of *Plasmodium* species relies on molecular beacon probe-based melting curve analysis. In addition, this assay might be used to quantify the parasitaemia of at least *P. falciparum* by calculating the level of parasitaemia directly from the Cq-value.

**Methods:**

The samples used in this study comprised reference samples, patient samples, and synthetic controls. The following analytical performance characteristics of the MC004 assay were determined: analytical specificity, limit of detection, the ability to detect mixed infections, and the potential to determine the level of parasitaemia of *P. falciparum*, including assessment of within-run and between-run precisions.

**Results:**

No false positive or false negative results were observed. The limit of detection of *P. falciparum* was 1 × 10^–3^ IU/mL (WHO standard). Mixed infections with *P. falciparum* and non-*falciparum* species were correctly identified. A calibration curve could be established to quantify the parasitaemia of at least *P. falciparum*. The within-run and between-run precisions were less than 20% CV at the tested parasitaemia levels of 0.09%, 0.16%, 2.15% and 27.27%.

**Conclusion:**

Based upon the analytical performance characteristics that were determined, the MC004 assay showed performance suitable for use in clinical settings, as well as epidemiological studies.

**Supplementary Information:**

The online version contains supplementary material available at 10.1186/s12936-021-03842-8.

## Background

The genus *Plasmodium* consists of over 200 widely distributed species, of which at least six are now known to regularly infect humans: *Plasmodium falciparum*,* Plasmodium vivax*,* Plasmodium malariae*,* Plasmodium ovale wallikeri*,* Plasmodium ovale curtisi*, *Plasmodium knowlesi* [1]. Previously, malaria was thought to be primarily caused by just the four species, *P. falciparum*, *P. vivax*, *P. malariae* and *P. ovale*, and only very rarely by other *Plasmodium* species. However, the entry of PCR-based techniques into malaria diagnostics has changed this view. It has led to the discovery of the existence of *P. ovale wallikeri* and *P. ovale curtisi* [2], and the demonstration that *P. knowlesi* is the predominant cause of malaria in regions of South East Asia. Interestingly, recent research indicates that also *P. cynomolgi* may be more common in South East Asia than previously suspected [3, 4]. In addition, human susceptibility to *Plasmodium simium* and *Plasmodium brasilianum* has been shown. *Plasmodium simium* is found throughout South America, while *Plasmodium brasilianum* seems restricted to a specific part of Brasil [5–7]. Globally, *P. falciparum* is responsible for the vast majority of malaria deaths and cases of severe malaria. Malaria caused by other *Plasmodium* species is not typically as life threatening, but can also be lethal. In particular, severe and fatal cases of malaria caused by *P. vivax* and *P. knowlesi* are well recognized [1].

The gold standard of malaria diagnosis is the examination of stained thick and thin blood films by light microscopy. The quantification of malaria parasites can be used to make clinical management decisions as well as monitoring response to treatment [8]. Microscopic diagnosis of malaria is prone to human error owing to its subjective nature. An inherent weakness of microscopy is the dependence on morphological features to distinguish *Plasmodium* species. Even under ideal conditions, reliable distinction of the infecting *Plasmodium* species can be very difficult, if not impossible. Particularly, *P. vivax* and *P. ovale* cannot always be easily differentiated based on morphology, distinguishing *P. knowlesi* from *P. malariae* can be very challenging, *P. ovale wallikeri* and *P. ovale curtisi* are morphologically identical, *P. cynomolgi* is morphologically indistinguishable from *P. vivax*, and *P. simium* and *P. brasilianum* cannot be distinguished by microscopy from *P. vivax* and *P. malariae*, respectively [9–11]. Also, the detection limit of microscopy is not always sufficient to detect low-density malaria infections. Submicroscopic infections are typically asymptomatic, but not benign. Such cases should be considered as chronic malaria, and should be treated accordingly. Untreated chronic malaria has significant health consequences for the individual, and, as it could potentially be transmitted, affects public health [12–15].

A plethora of polymerase chain reaction (PCR)-based nucleic acid amplification tests (NAATs) to detect, quantify and identify *Plasmodium* parasites in blood have been described in the literature. Rapid NAATs with a turnaround time of less than one hour are now available, and some NAATs can be used for high throughput of samples. In addition, NAATs are used to detect anti-malarial drug resistance markers. NAATs offer several significant advantages over standard microscopy. Importantly, the sensitivity of NAATs for the detection of *Plasmodium* parasites in blood is superior to any other diagnostic test. The general ability to detect far less than ten *Plasmodium* parasites per microlitre of blood provides the sensitivity to rule out malaria with high negative predictive value in clinical settings. In endemic settings, where the goal is to eradicate malaria, the required sensitivity to reliably detect submicroscopic infections can be met. Also, NAATs tend to be highly specific. Identification of *Plasmodium* species goes beyond what is possible with conventional microscopy. For example, for differentiating *Plasmodium* species with similar morphologies, detecting mixed *Plasmodium* infections, utilization of poor-quality or ambiguous samples, and when differentiation of *Plasmodium* species or strains is impossible based on morphology alone.

However, an important pitfall to be aware of is that NAATs not specifically designed to identify a certain *Plasmodium* species may fail to detect it or cause misidentification. Individual patient treatment may not always be affected, but such errors could ultimately prompt public health concerns by compromising malaria control and elimination.

Other concerns regarding the implementation of NAATs are the special equipment and reagents required, and the need for skilled laboratory technicians able to perform testing and to interpret the results of these relatively complex tests.

Furthermore, test characteristics vary widely, and although the World Health Organization (WHO) International Standard for *P. falciparum* [National Institute for Biological Standards and Control (NIBSC)-code 04/176] allows for comparison among the available assays, there is a lack of standardization and validation. As science and technology proceed, NAATs are bound to play an increasingly important role in malaria diagnosis, research, and epidemiology. At present, it is clear that NAATs cannot completely replace traditional microscopy [16–18].

The objective of the present study was to determine the analytical performance of the novel MC004 real-time PCR assay for malaria diagnosis (MRC Holland, Amsterdam, the Netherlands). This single-tube assay has been developed for the purpose of simultaneously detecting all *Plasmodium* species known to infect humans, and discrimination between *P. falciparum, P. vivax, P. malariae, P. ovale wallikeri, P. ovale curtisi*, *P. knowlesi* (including differentiation of three strains) and *P. cynomolgi* (including differentiation of three strains). In addition, this study shows how this assay might be used to estimate the parasitaemia of at least *P. falciparum*.

## Methods

### Analytical specificity study design

The analytical specificity of the assay was determined by testing samples received from reference institutes or referral laboratories [the Dutch Foundation for Quality Assessment in Medical Laboratories (SKML), UK NEQAS, Streeklab Haarlem—Laboratory for Medical Microbiology, the Biomedical Primate Research Centre, the London School of Hygiene & Tropical Medicine, and Labor Dr. Wisplinghoff]. In total, 40 reference samples were tested that included all seven *Plasmodium* species (*P. falciparum*, *P. vivax*, *P. knowlesi*, *P. malariae*, *P. cynomolgi*, *P. ovale wallikeri*, and *P. ovale curtisi*) at various levels of parasitaemia, and four different non-*Plasmodium* parasites: *Babesia microti*, *Loa loa*, *Trypanosoma brucei rhodesiensis*, and *Leishmania donovani infantum*. Specimen types included ethylenediaminetetraacetic acid (EDTA) whole blood, isolated DNA, and Giemsa-stained blood or bone marrow slides (Table 1). The results of the MC004 assay were compared with the results of the reference laboratory.

**Table 1:** Analytical specificity panel

Reference institute or referring laboratory	Description, if applicable the survey number is shown in parentheses	Specimen type	Level of parasitaemia
Dutch Foundation for Quality Assessment in Medical Laboratories (SKML) The Blood- and intestinal parasites scheme (n = 7)	*P. falciparum* (2019.1B)	Blood smear	1.9%
	*P. falciparum* (2019.3E)	EDTA whole blood	Unknown
	*P. falciparum* (2019.3F)	EDTA whole blood	Unknown
	*P. vivax* (2018.1G)	EDTA whole blood	Unknown
	*P. ovale* (2017.4B)	Blood smear	Unknown
	*P. knowlesi* (2016.3B)	Blood smear	4.2%
	*Trypanosoma brucei rhodesiensis* (2019.1A)	Blood smear	Not applicable
UK NEQAS (n = 3) The Blood parasitology scheme	*P. malariae* (5796)	Blood smear	Unknown
	*Loa Loa* (5211)	Blood smear	Not applicable
	*P. falciparum* + *P. vivax* (5844)	Blood smear	0.2%
UK NEQAS The Malaria molecular scheme (n = 8)	No parasites (5220)	EDTA whole blood	Not applicable
	No parasites (5222)	EDTA whole blood	Not applicable
	No parasites (5363)	EDTA whole blood	Not applicable
	No parasites (5364)	EDTA whole blood	Not applicable
	*P. vivax* (5365)	EDTA whole blood	Unknown
	*P. vivax* (5366)	EDTA whole blood	Unknown
	*P. falciparum* (5221)	EDTA whole blood	0.0002%
	*P. knowlesi* (5219)	EDTA whole blood	0.02%
Streeklab Haarlem—Laboratory for Medical Microbiology (n = 17)	*P. falciparum*	Blood smear	4.5%
	*P. falciparum*	Blood smear	1.3%
	*P. falciparum*	Blood smear	0.5%
	*P. falciparum*	Blood smear	9.9%
	*P. falciparum*	Blood smear	< 0.1%
	*P. falciparum*	Blood smear	Gametocytes
	*P. vivax*	Blood smear	Unknown
	*P. vivax*	Blood smear	Unknown
	*P. vivax*	Blood smear	Unknown
	*P. ovale*	Blood smear	Unknown
	*P. ovale*	Blood smear	Unknown
	*Leishmania donovani infantum*	Bone marrow smear	Not applicable
	*Leishmania donovani infantum*	Bone marrow smear	Not applicable
	No parasites	Blood smear	Not applicable
	No parasites	Blood smear	Not applicable
	*P. knowlesi*	Blood smear	Unknown
	*Babesia microti*	Blood smear	Not applicable
Biomedical Primate Research Centre (n = 2)	*P. knowlesi* (H strain)	Isolated DNA	Not applicable
	*P. cynomolgi* (M strain)	Isolated DNA	Not applicable
The London School of Hygiene & Tropical Medicine (n = 2)	*P. ovale wallikeri*	Isolated DNA	Not applicable
	*P. ovale curtisi*	Isolated DNA	Not applicable
Labor Dr. Wisplinghoff (n = 1)	*P. knowlesi*	EDTA whole blood	0.3%

### MC004 assay technology

The MC004 assay (MRC-Holland, Amsterdam, the Netherlands) is a multiplex nucleic acid amplification test that detects mitochondrial *Plasmodium* DNA encompassing the cyclo-oxygenase 3 (COX-3), cyclo-oxygenase 1 (COX-1) and cytochrome *b* (CYTB) genes from *Plasmodium* species that cause malaria in humans. The assay provides a qualitative result for the presence/absence of *Plasmodium* DNA, and discriminates between 11 *Plasmodium* species/strains (*P. falciparum*, *P. vivax*, *P. malariae*, *P. ovale wallikeri*, *P. ovale curtisi*, *P. knowlesi* LT48, *P. knowlesi* ATCC 30153, *P. knowlesi* ATCC 30158 *P. cynomolgi* ATCC 30149, *P. cynomolgi* KJ569866.1 and *P. cynomolgi* KJ569868.1). The MC004 assay involves two main steps, which take place in a single tube: (1) asymmetric target amplification by two different primer sets (primer pair 1: 5ʹ-TCGCTTCTAACGGTGAACT-3ʹ/5ʹ-AAGCAAACACTAGCGGTGGAA-3ʹ and primer pair 2: 5ʹ-CAGTATAATATTGTAATTTGATCAGTATGAG-3ʹ/5ʹ-GGATATTGTATAAATGATGCTATATCAGGTA-3ʹ). Primer pair 1 was designed to amplify a 116 base pair fragment of all 11 *Plasmodium* species/strains. Primer pair 2 was designed to amplify a 212 base pair fragment of *P. vivax*, *P. knowlesi*, and *P. cynomolgi* only. See Table 2 for the precise annealing positions of the primers. Amplification can be monitored real-time in the FAM detection channel due to the presence of a nonspecific green fluorescent nucleic acid dye that becomes fluorescent upon binding to double stranded DNA. (2) detection and differentiation of the amplicons by molecular beacon probe-based melting curve analysis. The assay uses three different probes, which are either Texas red-labelled (identical to the sequence of *P. knowlesi* ATCC 30153), Cy5-labelled (identical to the sequence of *P. falciparum*), or Cy5.5 labelled (identical to the sequence of *P. knowlesi* LT48).

**Table 2:** Annealing positions of the forward and reverse primers of primer pairs 1 and 2

*Plasmodium* species	GenBank accession number	Annealing positions of the forward and reverse primers of primer pair 1^a^	Annealing positions of the forward and reverse primers of primer pair 2^a^
*P. falciparum*	LR605957.1	Forward: 339–357 Reverse: 434–454	Not applicable
*P. vivax*	KY923424.1	Forward: 1493–1511 Reverse: 1588–1608	Forward: 1941–1971 Reverse: 2122–2152
*P. malariae*	AB489194.1	Forward: 1560–1578 Reverse: 1655–1675	Not applicable
*P. ovale wallikeri*	HQ712053.1	Forward: 1038–1056 Reverse: 1133–1153	Not applicable
*P. ovale curtisi*	HQ712052.1	Forward: 1038–1056 Reverse: 1133–1153	Not applicable
*P. knowlesi*	LR701176.1	Forward: 340–358 Reverse: 435–455	Forward: 781–811 Reverse: 962–992
*P. cynomolgi*	AB444125.1	Forward: 1605–1623 Reverse: 1700–1720	Forward: 2051–2081 Reverse: 2232–2262

See main text for the primer sequences. The expected amplicon sizes of primer pairs 1 and 2 are 116 and 212 base pairs, respectively

^a^According to the deposited sequences in GenBank under the shown accession numbers

### Specimen processing

The preferred specimen type is DNA extracted from 200 µL of (EDTA) human whole blood. DNA was isolated using the QIAamp DNA Blood Mini QIAcube Kit (Qiagen, Hilden, Germany) on a QIAcube instrument (Qiagen, Hilden, Germany) following the manufacturer’s specifications. DNA was eluted with 100 µL of elution buffer. Giemsa-stained blood smears were processed by pipetting 10 µL of 10 mM sodium hydroxide (NaOH pellets, 99%, Merck, Darmstadt, Germany) onto the surface of the blood smear. Subsequently, blood smear material was scraped off from the glass slide by making circular movements with a sterile lancet (Solofix, B Braun, Oss, Netherlands). The collected specimen was transferred to a sterile 2.0 mL tube (Eppendorf, Hamburg, Germany) that contained 100 µL of 10 mM sodium hydroxide. Processed specimen samples were stored at − 30 °C.

### Amplification and detection

Each reaction was carried out in a final volume of 25 µL reaction mixture containing 23 µL of MC004 master mix (MRC-Holland, Amsterdam, the Netherlands) and 2 µL of template. The components of the master mix were: both forward primers (1.25 µM), both reverse primers (0.25 µM), all three probes (0.5 µM), fluorescent compound, dNTPs, reaction buffer, and Taq DNA polymerase. The following settings were used for the amplification step: 95 °C for 3 min, followed by 50 cycles of 95 °C for 15 s, 60 °C for 30 s and 68 °C for 40 s. The following settings were used for the melting curve step: a gradual temperature increase from 25 to 69.4 °C (0.4 °C per 5 s).

### Controls

Positive and negative control samples were used to validate run validity. Each run included two negative controls: (1) with elution buffer added as template and (2) a fresh uninfected whole blood specimen processed as a separate sample. Positive control samples for each *Plasmodium* species come with the MC004 assay as provided by MRC Holland. The 1st WHO International Standard for *P. falciparum* DNA (NIBSC code: 04/176) diluted in EDTA whole blood (before DNA isolation) to a concentration of 1 × 10^–3^ IU/mL was used as a positive control for limit of detection. Each sample should produce an amplification curve, if no amplification curve is observed, this may be a sign of inhibition and renders the result invalid.

### Instrumentation

The assay was performed on the CFX96 Touch Real-Time PCR Detection System (Bio-Rad, Hercules, CA, USA). Runs were analysed using the accompanying Bio-Rad CFX manager 3.1 software (Bio-Rad, Hercules, CA, USA). Cq-values were determined by the software’s Cq Determination Mode (settings: Single Threshold set at 2000; Baseline Setting: Baseline Subtracted Curve Fit).

Melting temperatures were determined using the software’s default settings. The throughput is approximately 80 specimens for 1 operator in about 3.5 h. Up to 12 specimens can be processed, including DNA isolation using the QIAcube, within four hours of arrival of the specimens at the laboratory.

### Limit of detection study design

The limit of detection of the MC004 assay was determined by preparing a tenfold serial dilution of the WHO International Standard for *P. falciparum* DNA (NIBSC, Hertfordshire, England) in uninfected EDTA whole blood to achieve a concentration range of 1 × 10^9^ IU/mL to 1 × 10^–9^ IU/mL. To clarify, this WHO standard was assigned an arbitrary unitage in International Units (IU), unrelated to the level of parasitaemia or DNA copy number [19]. The lyophilized WHO standard, reconstituted in 500 µL of sterile water, was diluted in EDTA whole blood. Total volume of each dilution was 400 µL. DNA was isolated as described above, and samples were tested in three independent runs. The limit of detection was determined as the lowest concentration of *P. falciparum* detected in all three runs. In addition, similar tenfold serial dilutions of patient samples with known parasitaemia were tested: *P. falciparum* 1.3%*, P. falciparum* 0.2%, *P. vivax* < 0.1%*, P. malariae* 0.3%, *P. ovale curtisi* < 0.1%*,* and *P. knowlesi* ATCC 30158 0.3%. Samples were tested in two independent runs. The limit of detection was determined as the lowest concentration of the *Plasmodium* species detected in both runs.

### Detection of mixed infections study design

Mixed *Plasmodium* infections were created by adding together two DNA samples, each containing a different *Plasmodium* species (*P. falciparum* and a non-*falciparum* species) 1:1 (equal volume). When applicable, the parasitaemia was determined by experienced microscopists (*P. falciparum* 0.2%, *P. vivax* 0.1%, *P. ovale wallikeri* 0.2%, *P. malariae* 0.4%, *P. knowlesi* ATCC 30158 0.3%, *P. ovale curtisi* and *P. cynomolgi* KJ569868.1 isolated DNA diluted 100 times with sterile water). In addition, a two-fold serial dilution (1:2, 1:4, 1:8, 1:16, 1:32, 1:64, 1:128) of a *P. falciparum* DNA sample (initial parasitaemia was 1.3%) in sterile water was prepared to create mixed infections of *P. falciparum* and a non-*falciparum* species (non-*falciparum* samples as described above).

### Quantification of parasitaemia study design

To quantify parasite density, a calibration curve was established by plotting the microscopically determined parasite densities of ten samples from patients with *P. falciparum* infections as a function of the Cq-value. The parasite densities were: 0.1%, 0.1%, 0.4%, 0.8%, 1.3%, 1.5%, 3.7%, 23.7%, 31.8%, and 35.8%. Cq-values were determined in three independent runs. The exponential equation (y = a*e^(b*x)^) of the line of best fit through the 30 data points was determined using Microsoft Excel for Mac 2016, where ‘y’ is the parasitaemia (percentage of infected erythrocytes), ‘x’ is the Cq-value (threshold cycle), ‘a’ and ‘b’ are the dimensionless coefficients that define the equation, and ‘e’ is the base of the natural logarithm.

### Precision of the quantification of the parasitaemia study design

Within-run and between-run precisions [standard deviations (SD) and coefficients of variation (CV)] for the quantification of the parasitaemia were determined using four clinical samples infected with *P. falciparum*. The parasitaemia levels of these four samples were estimated by real-time PCR to be around 0.1%, 0.2%, 2% and 27%, respectively. Precision was assessed by testing five replicates per run over five runs. Each run the samples were tested in different wells of the PCR plate. Within-run and between-run precision were calculated as described by Chesher, 2008 [20].

## Results

### Melting temperatures and melting curves

**Fig. 1:**
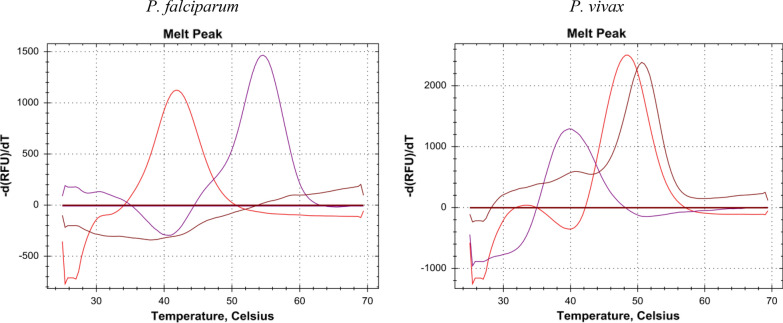
Representative melting curves of *P. falciparum* and *P. vivax*. The x-axis shows the temperature (°C). The y-axis shows the negative derivative of fluorescence (RFU) with respect to temperature (T). The *Plasmodium* species is indicated above each melting curve diagram. Red curves correspond to the Texas Red labelled probe, purple curves to the Cy5 labelled probe, and brown curves to the Cy5.5 labelled probe*.* For the sake of clarity, the melting temperature thresholds were manually set at zero

Probe melting temperatures for each *Plasmodium* species/strain are shown in Table 3. The observed maximum deviation of melting points was 1.2 °C. Figure 1 shows representative melting curves of *P. falciparum* and *P. vivax* (see Additional file 1: Figure S1 for the melting curves of all other *Plasmodium* species/strains).

**Table 3:** Melting temperatures of the Texas Red, Cy5 and Cy5.5 Labelled probes for 11 *Plasmodium* species/strains

*Plasmodium* species	Melting temperature (°C) Texas Red labelled probe	Melting temperature (°C) Cy5 labelled probe	Melting temperature (°C) Cy5.5 labelled probe
*P. falciparum*	42.4	54.4	None
*P. vivax*	49.2	40.4	51.6
*P. malariae*	39.6	33.6	None
*P. ovale wallikeri*	49.6	48.4	None
*P. ovale curtisi*	53.2	48.4	None
*P. knowlesi* LT48	49.2	40.8	62.8
*P. knowlesi* ATCC 30158	56.4	40.8	62.8
*P. knowlesi* ATCC 30153	56.4	40.8	52.4
*P. cynomolgi* ATCC 30149	56.0	40.4	57.6
*P. cynomolgi* KJ569866.1	56.4	40.8	54.8
*P. cynomolgi* KJ569868.1	50.8	40.8	58.8

Differences in probe melting temperatures enable distinction between the 11 *Plasmodium* species/strains

### Analytical specificity

Testing of samples that did not contain any parasites and samples that contained *Leishmania donovani infantum*, *Loa loa*, *Trypanosoma brucei rhodesiensis*, or *Babesia microti*, yielded no false positive results. Among the samples that did contain *Plasmodium* parasites, one discrepancy was observed between the result of the reference institute [see Table 1: SKML, *P. vivax* (2018.1G)] and the MC004 assay (*P. knowlesi*). This sample was further analysed by Sanger sequencing, and this confirmed the presence of *P. knowlesi*. No data regarding the origin of this specimen were available at the reference institute, therefore leaving the question of what might explain this anomaly open-ended. The analytical specificity panel contained one *P. cynomolgi* sample, which showed melting temperatures consistent with *P. cynomolgi* KJ569868.1. Five of the six *P. knowlesi* samples showed melting temperatures consistent with *P. knowlesi* LT48, the other one with *P. knowlesi* ATCC 30158. The one included mixed infection (UK NEQAS 5844, *P. falciparum* + *P. vivax*) was readily identified. Thus, among the 40 samples that were included in the specificity panel, no false positive or false negative results were observed with the MC004 assay.

### Limit of detection

Limit of detection results are presented in Table 4. The 1st WHO International Standard for *P. falciparum* DNA (NIBSC code: 04/176) was detectable until a dilution of 10^–12^, which corresponds to a concentration of 1 × 10^–3^ IU/mL. The undiluted concentration of this standard has been described to correspond to 9.79 parasites per 100 red blood cells [21]. However, arguably, expressing the limit of detection as number of parasites per volume of blood would be misrepresentative, as the limit of detection determined by dilution series appears to be dependent on the starting concentration. Illustrative of this point are the dilutions of *P. falciparum* with a relatively high parasitaemia of 1.3%, which was still detectable at dilution 10^–14^, and of *P. falciparum* with a relatively low parasitaemia of 0.2%, which was detectable until dilution 10^–5^. Limits of detection of *P. vivax*, *P. knowlesi* ATCC 30158, and *P. ovale curtisi* were determined at dilution 10^–4^, that of *P. malariae* at dilution 10^–7^. Other *Plasmodium* species/strains were not tested due to lack of sample.

**Table 4:** Limit of detection

*Plasmodium* species	Undiluted parasite concentration	Highest dilution that consistently detected the *Plasmodium* species
*P. falciparum* (NIBSC)	1 × 10^9^ IU/mL	10^–12^ (1 × 10^–3^ IU/mL)
*P. falciparum*	1.3%	10^–14^ (the highest dilution that was tested)
*P. falciparum*	0.2%	10^–5^
*P. vivax*	< 0.1%	10^–4^
*P. malariae*	0.3%	10^–7^
*P. ovale curtisi*	< 0.1%	10^–4^
*P. knowlesi* ATCC 30158	0.3%	10^–4^

### Detection of mixed infections

**Fig. 2:**
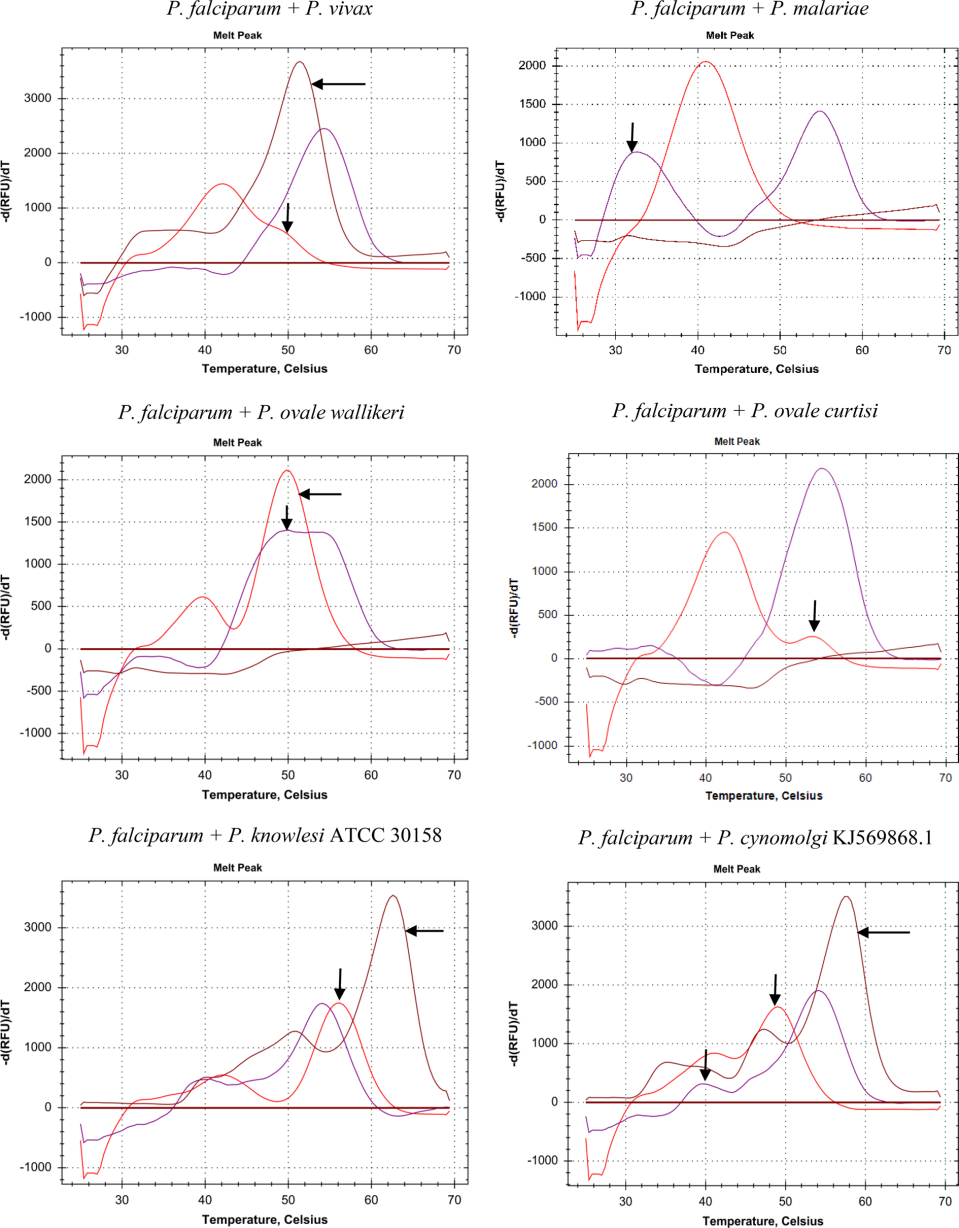
Melting curves of mixed *Plasmodium* infections (*P. falciparum* with non-*P. falciparum*). The x-axis shows the temperature (°C). The y-axis shows the negative derivative of fluorescence (RFU) with respect to temperature (T). The type of mixed infection is indicated above each melting curve diagram. Red curves correspond to the Texas Red labelled probe, purple curves to the Cy5 labelled probe, and brown curves to the Cy5.5 labelled probe. The arrows indicate the differences between a mixed infection and a mono-infection with P. falciparum. For the sake of clarity, the melting temperature thresholds were manually set at zero

The melting curves of the created mixed *Plasmodium* infections at parasitaemia levels ranging from 0.1 to 0.4% (*P. falciparum* with either *P. vivax*, *P. ovale wallikeri*, *P. ovale curtisi*, *P. knowlesi* ATCC 30158, or *P. cynomolgi* KJ569868.1) are shown in Fig. 2. All tested mixed *Plasmodium* infections could be visually identified by the presence of more than one melting peak in the Texas Red and/or Cy5 channels, shifted melting temperatures, a shoulder added to the original melting peak, or the presence of a Cy5.5 melting peak.

The melting curves of the created mixed *Plasmodium* infections using dilution 1:128 of *P. falciparum* (initial parasitaemia level of 1.3%) with either *P. vivax*, *P. ovale wallikeri*, *P. ovale curtisi*, *P. knowlesi* ATCC 30158, or *P. cynomolgi* KJ569868.1 are shown in Additional file 2: Figure S2. Mixed infections with *P. vivax*, *P. ovale wallikeri*, and *P. ovale curtisi* could be visually identified. Mixed infections with *P. malariae*, *P. knowlesi* ATCC 30158, and *P. cynomolgi* KJ569868.1 could be readily identified up to dilution 1:64. In these mixed infections, *P. falciparum* could just be detected at dilution 1:128 by a small melting peak of the Cy5-labelled probe.

### Quantification of parasitaemia (*P. falciparum*)

**Fig. 3:**
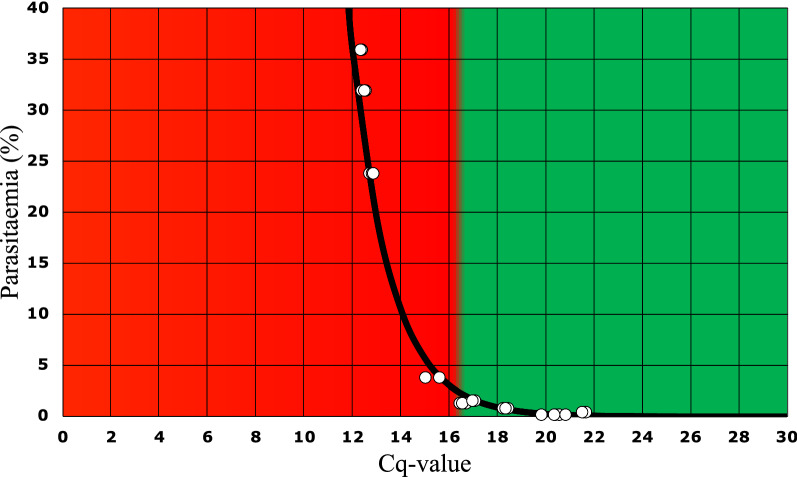
The calibration curve for calculating parasitaemia from Cq-value. By microscopy estimated *P. falciparum* parasitaemia (y-axis) is plotted as a function of the Cq-value (x-axis). The black curve through the white data points (see also Additional file 3: Table S1) is the exponential line of best fit with the equation: parasitaemia equals 57,012 * e^(−^ ^0.615*Cq-value)^. The red part of the figure indicates parasitaemia > 2% (Cq-values less than 16.68); the green part of the figure indicates parasitaemia of < 2% (Cq-values greater than 16.68)

The 30 Cq-values used to construct the calibration curve are shown in Additional file 3: Table S1. The calibration curve itself is shown in Fig. 3. The exponential line of best fit has the equation: parasitaemia equals 57,012 * e^(− 0.615*Cq-value)^. Figure 3 illustrates that variation in Cq-values in the lower range has a greater impact than variation in Cq-values in the higher range with regard to the calculated parasitaemia. For example, the difference between the calculated parasitaemia from a Cq-value of 15 (5.6%) and 16 (3.0%) is 2.6%, while the difference between the calculated parasitaemia from a Cq-value of 20 (0.26%) and 21 (0.14%) is 0.12%. The most commonly used cut-off in clinical practice is a parasitaemia level of 2%, which corresponds to a Cq-value of 16.68.

### Precision of the quantification of the parasitaemia (*P. falciparum*)

The results of the within-run and between-run precisions are summarized in Table 5. The within-run and between-run precisions at level 1 (average parasitaemia of 27.27%) were 4.6% and 7.8% CV, respectively. The between-run precisions equaled the within-run precision at level 3, 4 and 5 (average parasitaemia of 2.15%, 0.16% and 0.09%, respectively). This indicates stability under changing conditions.

**Table 5:** Within-run and between-run precisions of *P. falciparum* parasitaemia determined using the MC004 assay

Level	Average *P. falciparum* parasitaemia per level (%)	Within-run precision SD/CV	Between-run precision SD/CV
1	27.27	1.257/4.6	2.133/7.8
2	2.15	0.363/16.9	0.363/16.9
3	0.16	0.022/13.6	0.022/13.6
4	0.09	0.012/14.4	0.012/14.4

Five replicates per run were tested over five runs at four levels of parasitaemia. Parasitaemia was calculated from the Cq-value using the equation of the calibration curve (Fig. 3)

*SD* standard deviation, *CV* coefficient of variation

## Discussion

### Analytical specificity

No cross-reactivity was observed in the MC004 assay between the evaluated *Plasmodium* and non-*Plasmodium* species. The MC004 assay has been designed to specifically amplify *Plasmodium* DNA, and includes post-PCR melting curve analysis based on three different molecular beacon probes to determine specificity of the amplicons. Therefore, the occurrence of false-positive results in case of non-*Plasmodium* infections is deemed to be a highly unlikely possibility. This presumption is supported by the fact that cross-reactivity issues between *Plasmodium* and non-*Plasmodium* species have not been raised as a concern in previously published studies. However, cross-reactivity between different *Plasmodium* species is a well-known issue. In particular, the nucleotide sequences of *P. vivax* and *P. knowlesi* share high similarity, preventing specific PCR amplification in some assays. The same holds true for *P. ovale curtisi* and *P. ovale wallikeri*. This study shows these issues of cross-reactivity have been resolved by the MC004 assay. Arguably, cross-reactivity is the most likely explanation for the one discrepant result found with the MC004 assay (See Table 1, *P. vivax* (SKML sample 2018.1G). The result of MC004 was *P. knowlesi*, which was confirmed by Sanger sequencing), but it may also have been a clerical error or an oversight. The differentiation of *P. knowlesi* ATCC 30158 and *P. knowlesi* LT48 was verified, but no sample was available that contained *P. knowlesi* ATCC 30153. The only available *P. cynomolgi* sample showed melting temperatures consistent with strain KJ569868.1. Each *Plasmodium* species could be straightforwardly identified using the melting temperatures shown in Table 3. The MC004 assay cannot differentiate between *P. vivax* and *P. simium*, as their mitochondrial genomes differ at two nucleotides positioned outside the regions covered by the probes. Also, the genomes of *P. malariae* and *P. brasilianum* are very similar, and will have identical melting temperatures [22]. In clinical practice, this would not affect patient management. To reduce the probability of human error and to increase the speed of reporting results, species identification could be performed and reported automatically by programming the laboratory information management system, or semi-automatically by programming software like Microsoft Excel to return the species after entering the melting points.

### Limit of detection

The MC004 assay was capable of detecting 1 × 10^–3^ IU/mL of the 1st WHO International Standard for *P. falciparum* DNA (NIBSC code: 04/176), which is equivalent to a dilution of 10^–12^. The dilution series of two *P. falciparum*-infected whole blood samples (parasitaemia of 0.2% and 1.3%) shows that the limit of detection determined this way depends on the starting concentration (Table 4). Therefore, the detection limit was not expressed as number of parasites per volume of blood, but rather as the highest dilution of an arbitrary, relatively low, undiluted parasitaemia at which the *Plasmodium* species was consistently detected. Since there is no WHO standard available for other *Plasmodium* species than *P. falciparum*, a dilution series of clinical samples was used to assess the limit of detection for other *Plasmodium* species. Both the undiluted parasitaemia, ranging from < 0.1% to 0.3%, and the highest dilutions at which *Plasmodium* DNA was still detected, ranging from 10^–4^ to 10^–7^, were roughly comparable between *P. falciparum*, *P. vivax*, *P. malariae*, *P. ovale curtisi* and *P. knowlesi* ATCC 30158. No clinical sample was available to assess the limit of detection of *P. cynomolgi*. These limits of detection are regarded as sufficient to warrant further testing in both clinical and research settings.

### Detection of mixed infections

Infections with more than one *Plasmodium* species are relatively rare in clinical practice, but relevant. Due to lack of sample, there was no opportunity to test actual samples from patients, besides the UK NEQAS sample (*P. falciparum* + *P. vivax*, See Table 1). Thus, mixed infections were mimicked by mixing DNA-isolates of *P. falciparum* and either *P. vivax*, *P. ovale wallikeri*, *P. ovale curtisi*, *P. knowlesi* ATCC 30158, or *P. cynomolgi* KJ569868.1. To further demonstrate the proof of principle that the MC004 assay can detect mixed infections, the amount of DNA of each *Plasmodium* species was kept roughly comparable, which was in the range of 0.1 to 0.4% parasitaemia (Fig. 2). An additional experiment, in which *P. falciparum* (with initial parasitaemia of 1.3%) was diluted up to 1:128, indicated that mixed infections may be missed if differences in DNA concentration between *Plasmodium* species are large (Additional file 2: Figure S2). *P. falciparum* is the most commonly identified species in our geographic area, and poses the greatest threat globally. Therefore, the focus was on mixed infections with *P. falciparum*, although any combination of two or even three *Plasmodium* species is theoretically possible. To what extent mixed infections are still detectable in cases where the difference between the *Plasmodium* species in parasitaemia are larger, or in case of infections with three *Plasmodium* species, remains to be verified by further studies, preferably empirically in the field. Features of the MC004 assay that specifically help to distinguish mixed infections with *P. falciparum* are the relatively large differences in melting temperatures of the Texas red and Cy5 labelled probes between *P. falciparum* and the other *Plasmodium* species, as well as the absence of a Cy5.5 melting temperature, which is only present in case of *P. vivax*, *P. knowlesi* or *P. cynomolgi* infections (Table 3).

### Quantification of parasitaemia (*P. falciparum*)

One of the medical decision points to identify patients with severe or complicated malaria, particularly *P. falciparum* malaria, is more than two percent of the red blood cells parasitized [23, 24]. This arguably conservative threshold has traditionally been estimated by microscopy and is used rather than the WHO criterion of a parasitaemia estimate of greater than 4% [25]. A means to use the MC004 assay to estimate the level of parasitaemia based on the correlation between Cq-value and amount of *Plasmodium* DNA was sought out. The resulting calibration curve is shown in Fig. 3, which is traceable to by microscopy estimated levels of parasitaemia. To spare clinical sample and for sake of standardization, the WHO International Standard for *P. falciparum* DNA could be used to reestablish or verify the calibration curve when appropriate by assigning parasitaemia values to Cq-values. The performance of the MC004 assay when put into practice to estimate the parasitaemia of both *P. falciparum* and non-*falciparum* species at various levels and at different developmental stages, is to be shown empirically.

### Precision of the quantification of the parasitaemia (*P. falciparum*)

The within-run and between-run precisions close to the most relevant medical decision point of 2% parasitaemia (2.15%) and in the lower range (0.16% and 0.09%) and high range (27.27%) of less than 20% CV indicates robustness of the MC004 assay. While consistent results were obtained between runs and instruments, only one technician performed the testing, and one reagent lot MC004 was used. Nevertheless, considering the generally large variation in parasitaemia estimates by microscopy, precision is expected to improve with the MC004 assay.

### Summary of limitations

This PCR protocol has limitations. First, differentiation between *P. vivax* and *P. simium* and between *P. malariae* and *P. brasilianum* is not possible, as the respective melting curves would be identical. Second, the detection of minority parasite populations in mixed infections may be compromised, if the differences in DNA concentration between *Plasmodium* species are too large. A methodological limitation of this study was the lack of field-collected parasites of each *Plasmodium* species or strain the PCR was designed to detect and identify, and of field-collected mixed infections.

## Conclusions

The MC004 assay is a novel single-tube real-time PCR assay developed for the purpose of simultaneously detecting all *Plasmodium* species known to infect humans, and discrimination between *P. falciparum*,* P. vivax, P. malariae*,* P. ovale wallikeri*, *P. ovale curtisi*, *P. knowlesi* (including differentiation of three strains) and *P. cynomolgi* (including differentiation of three strains). This study shows there is sufficient evidence of validity and reliability to warrant further testing of the MC004 assay to detect, identify, and quantify *Plasmodium* species causing malaria in humans in both clinical and research settings. A separate paper reports on the application of the MC004 assay in clinical practice (Bergen et al. pers. commun.).

## Supplementary Information

**Additional file 1: Figure S1.** Representative melting curves of *P. ovale curtisi, P. ovale wallikeri, P. malariae, P. knowlesi strains and P. cynomolgi strains.* The x-axis shows the temperature (°C). The y-axis shows the negative derivative of fluorescence (RFU) with respect to temperature (T). Red curves correspond to the Texas Red labelled probe, purple curves to the Cy5 labelled probe, and brown curves to the Cy5.5 labelled probe. For the sake of clarity, the melting temperature thresholds were manually set around -1000 RFU. Note that only *P. vivax, P. knowlesi* and *P. cynomolgi* show melting peaks of Cy5.5 labelled probes*.*

**Additional file 2: Figure S2.** Melting curves of mixed *Plasmodium* infections: *P. falciparum* (diluted 1:128) with non-*P. falciparum*. The x-axis shows the temperature (°C). The y-axis shows the negative derivative of fluorescence (RFU) with respect to temperature (T). The type of mixed infection is indicated above each melting curve diagram. Red curves correspond to the Texas Red labelled probe, purple curves to the Cy5 labelled probe, and brown curves to the Cy5.5 labelled probe. The arrows indicate the melting peaks of *P. falciparum*. Melting peaks not indicated are of the non-*P. falciparum* species. For the sake of clarity, the melting temperature thresholds were manually set at zero.

**Additional file 3: Table S3.** The 30 Cq-values used to construct the calibration curve and the corresponding parasitaemia of *P. falciparum* as estimated by microscopy.

## Data Availability

All datasets are presented in the manuscripts. Raw data of the PCR-runs and patient materials are not publicly available due to patient privacy concerns, but on reasonable request to the corresponding author every effort will be made to answer the questions raised.
