# Supplementary material for: Evaluation of a novel real-time PCR assay for the detection, identification and quantification of Plasmodium species causing malaria in humans
Source: Malar J. 2021 Jul 12;20:314. doi: 10.1186/s12936-021-03842-8 (PMC8274047; doi:10.1186/s12936-021-03842-8)

P. ovale curtisi

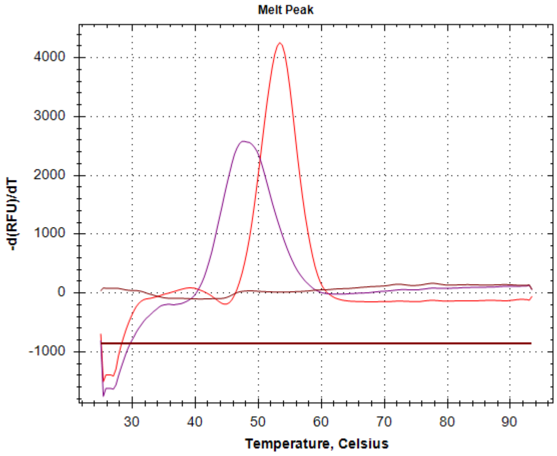

P. ovale wallikeri

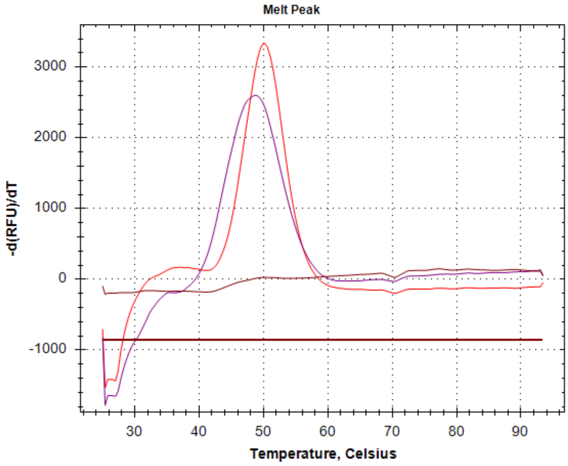

P. malariae

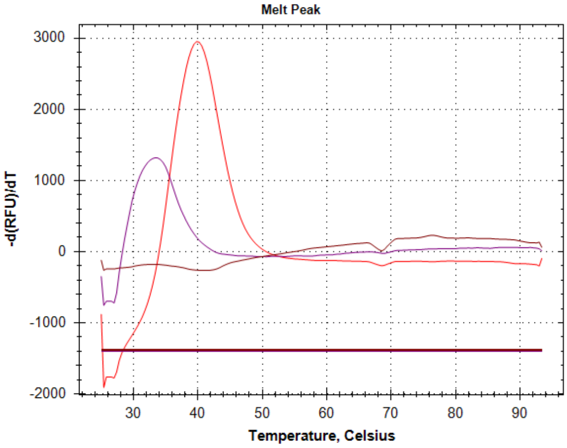

P. knowlesi LT48

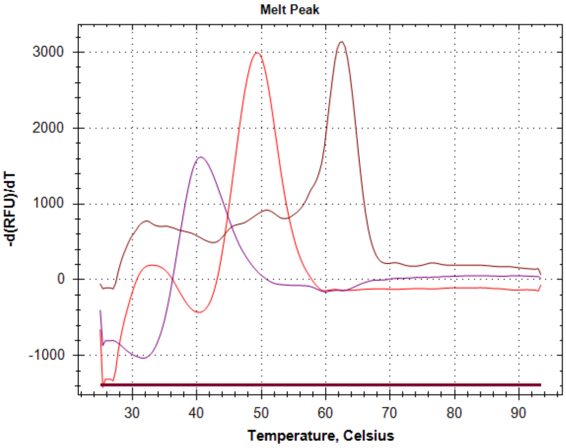

P. knowlesi ATCC 30153

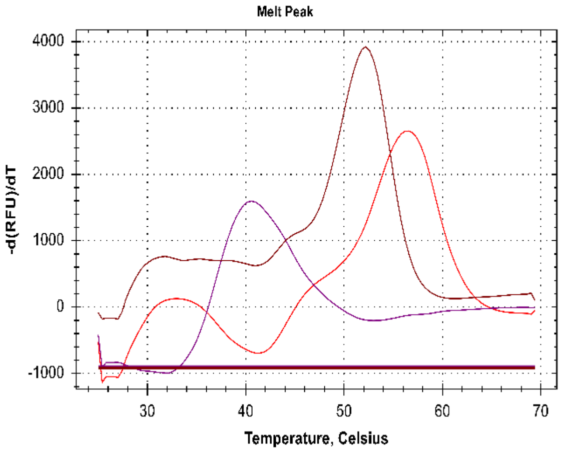

P. knowlesi ATCC 30158

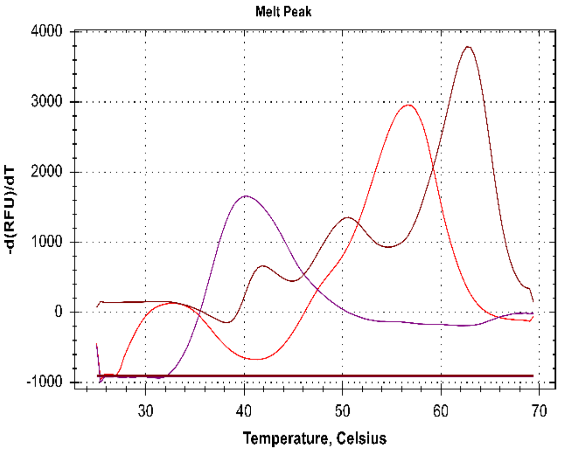

P. cynomolgi KJ 569868.1

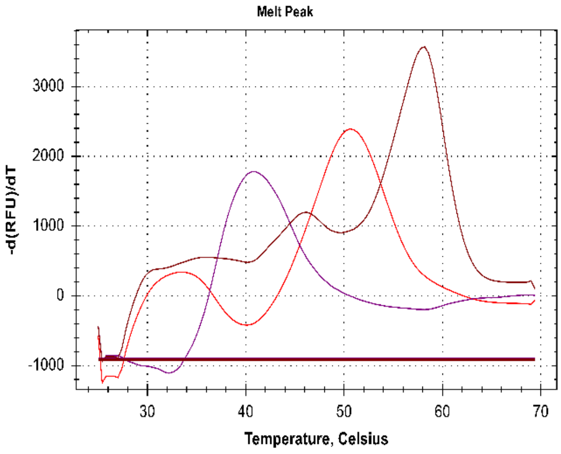

P. cynomolgi KJ 569866.1

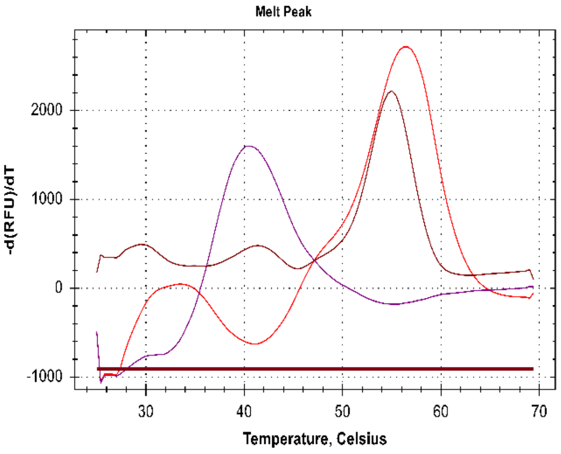

P. cynomolgi ATCC 30149

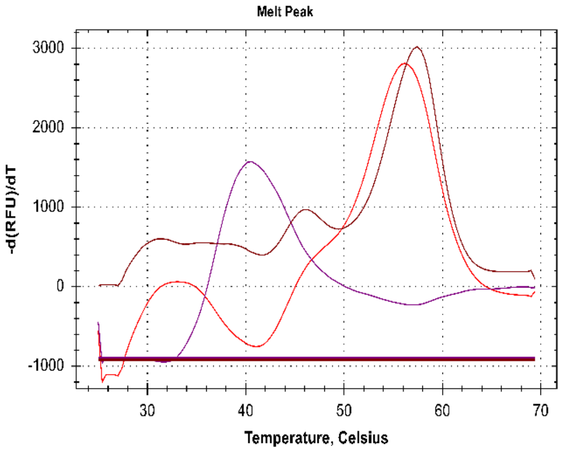

Supplement: Supplementary file 1 — Additional file 1: Figure S1. Representative melting curves of P. ovale curtisi, P. ovale wallikeri, P. malariae, P. knowlesi strains and P. cynomolgi strains. The x-axis shows the temperature (°C). The y-axis shows the negative derivative of fluorescence (RFU) with respect to temperature (T). Red curves correspond to the Texas Red labelled probe, purple curves to the Cy5 labelled probe, and brown curves to the Cy5.5 labelled probe. For the sake of clarity, the melting temperature thresholds were manually set around -1000 RFU. Note that only P. vivax, P. knowlesi and P. cynomolgi show melting peaks of Cy5.5 labelled probes. [file 12936_2021_3842_MOESM1_ESM.pdf]
