# Supplementary material for: Evaluation of a novel real-time PCR assay for the detection, identification and quantification of Plasmodium species causing malaria in humans
Source: Malar J. 2021 Jul 12;20:314. doi: 10.1186/s12936-021-03842-8 (PMC8274047; doi:10.1186/s12936-021-03842-8)

P. falciparum + *P. vivax*

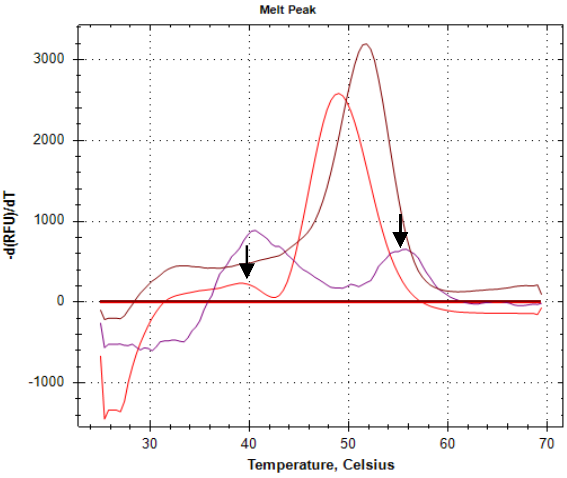

P. falciparum + *P. malariae*

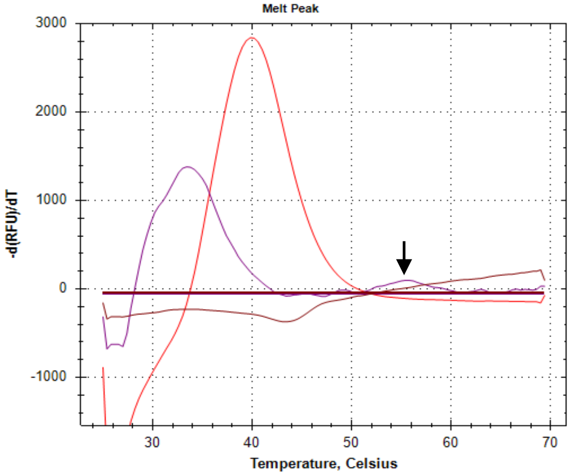

P. falciparum + *P. ovale wallikeri*

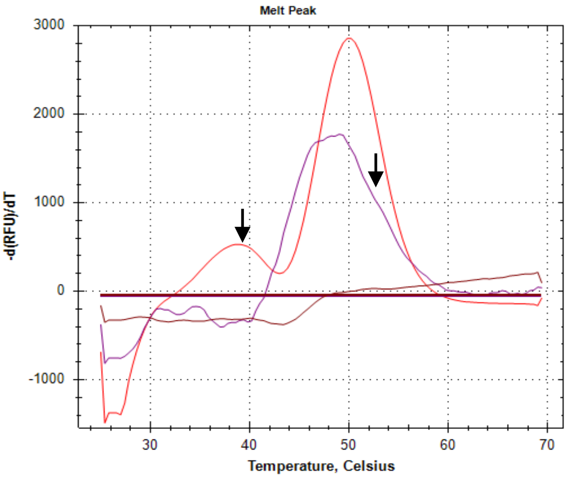

P. falciparum + *P. ovale curtisi*

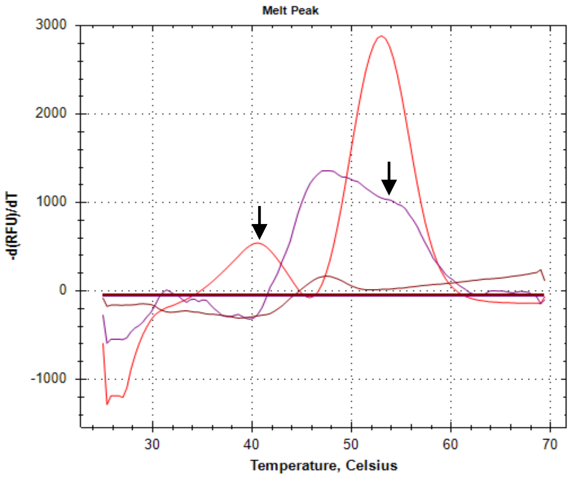

P. falciparum + *P. knowlesi* ATCC 30158

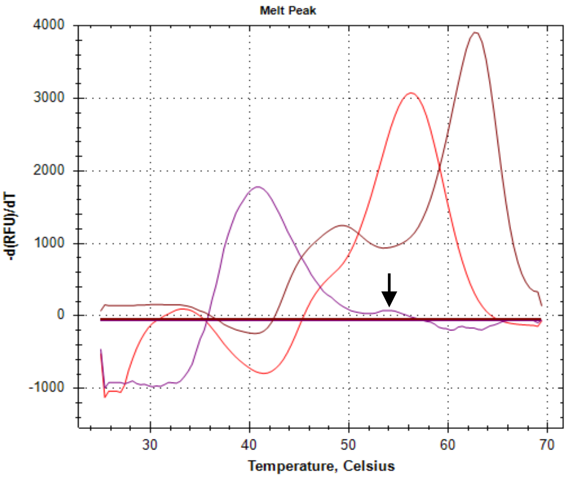

P. falciparum + *P. cynomolgi* KJ569868.1

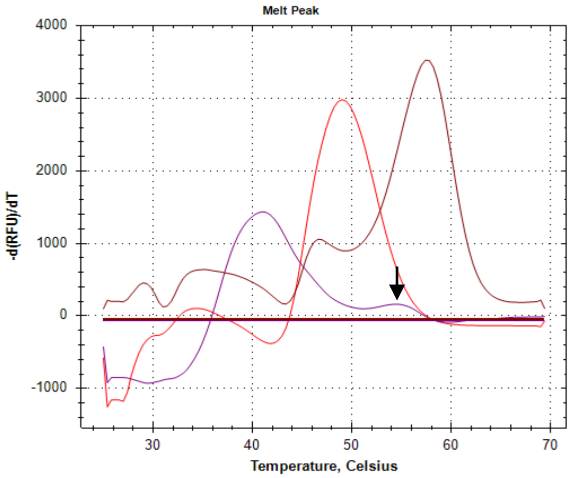

Supplement: Supplementary file 2 — Additional file 2: Figure S2. Melting curves of mixed Plasmodium infections: P. falciparum (diluted 1:128) with non-P. falciparum. The x-axis shows the temperature (°C). The y-axis shows the negative derivative of fluorescence (RFU) with respect to temperature (T). The type of mixed infection is indicated above each melting curve diagram. Red curves correspond to the Texas Red labelled probe, purple curves to the Cy5 labelled probe, and brown curves to the Cy5.5 labelled probe. The arrows indicate the melting peaks of P. falciparum. Melting peaks not indicated are of the non-P. falciparum species. For the sake of clarity, the melting temperature thresholds were manually set at zero. [file 12936_2021_3842_MOESM2_ESM.pdf]
