# Supplementary material for: Evaluation of a novel real-time PCR assay for the detection, identification and quantification of Plasmodium species causing malaria in humans
Source: Malar J. 2021 Jul 12;20:314. doi: 10.1186/s12936-021-03842-8 (PMC8274047; doi:10.1186/s12936-021-03842-8)
Supplement: Supplementary file 3 — Additional file 3: Table S3. The 30 Cq-values used to construct the calibration curve and the corresponding parasitaemia of P. falciparum as estimated by microscopy. [file 12936_2021_3842_MOESM3_ESM.docx]

**Additional files**

**Table S3. The 30 Cq-values used to construct the calibration curve and the corresponding parasitaemia of *P. falciparum* as estimated by microscopy.**

| **Parasitaemia estimated by microscopy (%)** | **Cq-value**  **PCR run 1** | **Cq-value**  **PCR run 2** | **Cq-value**  **PCR run 3** |
| --- | --- | --- | --- |
| 35.8 | 12.38 | 12.36 | 12.42 |
| 31.8 | 12.50 | 12.44 | 12.56 |
| 23.7 | 12.90 | 12.86 | 12.74 |
| 3.7 | 15.06 | 15.63 | 15.64 |
| 1.5 | 17.02 | 17.08 | 17.00 |
| 1.3 | 16.59 | 16.45 | 16.74 |
| 0.8 | 18.35 | 18.47 | 18.29 |
| 0.4 | 21.52 | 21.69 | 21.57 |
| 0.1 | 20.37 | 20.83 | 20.60 |
| 0.1 | 20.35 | 19.85 | 20.41 |
